# A rare case of unilateral adrenal hyperplasia accompanied by hypokalaemic periodic paralysis caused by a novel dominant mutation in *CACNA1S*: features and prognosis after adrenalectomy

**DOI:** 10.1186/1471-2490-14-96

**Published:** 2014-11-28

**Authors:** Bo Yang, Yuan Yang, Wenling Tu, Ying Shen, Qiang Dong

**Affiliations:** Department of Urology, West China Hospital, Sichuan University, Chengdu, 610041 China; Department of Medical Genetics, West China Hospital, Sichuan University, Chengdu, 610041 China

**Keywords:** Unilateral adrenal hyperplasia, Hypokalaemic periodic paralysis, *CACNA1S*, Adrenalectomy

## Abstract

**Background:**

Acute hypokalaemic paralysis is characterised by acute flaccid muscle weakness and has a complex aetiological spectrum. Herein we report, for the first time, a case of unilateral adrenal hyperplasia accompanied by hypokalaemic periodic paralysis type I resulting from a novel dominant mutation in *CACNA1S*. We present the clinical features and prognosis after adrenalectomy in this case.

**Case presentation:**

A 43-year-old Han Chinese male presented with severe hypokalaemic paralysis that remitted after taking oral potassium. The patient had suffered from periodic attacks of hypokalaemic paralysis for more than 20 years. A computed tomography (CT) scan of the abdomen showed a nodular mass on the left adrenal gland, although laboratory examination revealed the patient had not developed primary aldosteronism. The patient underwent a left adrenalectomy 4 days after admission, and the pathological examination further confirmed a 1.1 cm benign nodule at the periphery of the adrenal gland. Three months after the adrenalectomy, a paralytic attack recurred and the patient asked for assistance from the Department of Medical Genetics. His family history showed that two uncles, one brother, and a nephew also had a history of periodic paralysis, although their symptoms were milder. The patient’s *CACNA1S* and *SCN4A* genes were sequenced, and a novel missense mutation, c.1582C > T (p.Arg528Cys), in *CACNA1S* was detected. Detection of the mutation in five adult male family members, including three with periodic paralysis and two with no history of the disease, indicated that this mutation caused hypokalaemic periodic paralysis type I in his family. Follow-up 2 years after adrenalectomy showed that the serum potassium concentration was increased between paralyses and the number and severity of paralytic attacks were significantly decreased.

**Conclusion:**

We identified a novel dominant mutation, c.1582C > T (p.Arg528Cys), in *CACNA1S* that causes hypokalaemic periodic paralysis. The therapeutic effect of adrenalectomy indicated that unilateral adrenal hyperplasia might make paralytic attacks more serious and more frequent by decreasing serum potassium. This finding suggests that the surgical removal of hyperplastic tissues might relieve the symptoms of patients with severe hypokalaemic paralysis caused by other incurable diseases, even if the adrenal lesion does not cause primary aldosteronism.

## Background

Adrenal hyperplasia (AH) is a common endocrine disease, and unilateral adrenal hyperplasia (UAH) is a common cause of primary aldosteronism (PA) [1, 2]. Muscular paralysis and decreased serum potassium are two of the initial symptoms in some patients with PA-causing UAH, which can be corrected by adrenalectomy [3, 4]. However, it is unclear whether adrenalectomy in patients with non-PA-causing UAH increases serum potassium, which would indicate that this surgery could relieve symptoms caused by hypokalaemia resulting from other incurable diseases.

Hypokalaemic periodic paralysis (HOKPP), including HOKPP1 (OMIM #170400) and HOKPP2 (OMIM #613345), is a rare autosomal dominant inherited disease characterised by episodic muscle weakness with significant hypokalaemia (< 0.9 to 3.0 mmol/L) during attacks [5, 6]. HOKPP1 and HOKPP2 are caused by mutations in *CACNA1S* (calcium channel, voltage-dependent, L type, alpha 1S subunit) (OMIM #114208) and *SCN4A* (sodium channel, voltage-gated, type IV, alpha subunit) (OMIM #603967), respectively [7, 8]. The frequency of HOKPP attacks varies from daily to yearly, and the attacks typically last from 3–4 hours up to a day or longer [9]. The frequency of attacks varies significantly among members of families with HOKPP, and the cause for this variation remains unknown.

In this case, we report the clinical features, diagnosis, and prognosis after adrenalectomy in a patient with non-PA-causing UAH and HOKPP and discuss the potential of this surgery to relieve the symptoms of hypokalaemic periodic paralysis.

## Case presentation

A 43-year-old male presented to the outpatient Department of Urology of West China Hospital in March 2012 with severe paralytic attacks characterised by palpitations and muscle weakness starting in the right thigh and spreading to all limbs. A significant reduction in the serum potassium concentration (1.89 mmol/L, reference value 3.5–5.0 mmol/L) was found during laboratory examination, and an ECG indicated severe potassium deficiency. Symptoms remitted after taking oral potassium (50 ml of 10% potassium chloride was administered immediately followed by an additional 50 ml over 24 h for a total dose of 10 g). After the paralytic attack, a CT scan of the abdomen was performed, which revealed left UAH characterised by a nodular mass on the left adrenal gland. Laboratory examination showed a slight elevation in norepinephrine (602 ng/L, reference value, 272–559 ng/L) and a reduction in adrenaline (< 25 ng/L, reference value 54–122 ng/L) in the serum. Other data, including the serum concentrations of potassium (3.71 mmol/L), aldosterone (11.41 ng/dL, reference value 9.8–27.5 ng/dl), cortisol (7.3 μg/dL, reference value 7.2–18.2 μg/dL), renin (2.22 ng/mL, reference value 0.56–2.79 ng/ml), calcium (2.27 mmol/L, reference value 2.1–2.7 mmol/L), creatine kinase (58 IU/L, reference value 19–226 IU/L), lactate dehydrogenase (167 IU/L, reference value 110–220 IU/L), alanine aminotransferase (19 IU/L, reference value <55 IU/L), aspartate transaminase (15 IU/L, reference value <46 IU/L), creatinine (74.5 μmol/L, reference value 53–140 μmol/L), blood urea nitrogen (7.69 mmol/L, reference value 3.30–8.22 mmol/L), thyroid-stimulating hormone (6.3 mU/L, reference value 2–10 mU/L), total-triiodothyronine (2.14 nmol/L, reference value 1.8–2.9 nmol/L), and total thyroxine (87 nmol/L, reference value 65156 nmol/L), were normal. The patient did not have hypertension (117/83 mm Hg).

Patient history showed that the paralytic attacks were usually triggered by physical labour or stress and were periodic. Attack frequency varied from weekly during the summer to bimonthly in the winter; each attack lasted 4–6 hours. This attack was the most severe of the attacks he had experienced during the past decade. Although these paralytic attacks were associated with hypokalaemia, the aetiology had not been previously established, and the patient had not received any treatment, including potassium supplement, between attacks. Two years before this attack, serum potassium had been measured several times between attacks; the results of three of these tests were available and were 3.74, 3.69 and 3.63 mmol/L.

**Figure 1:**
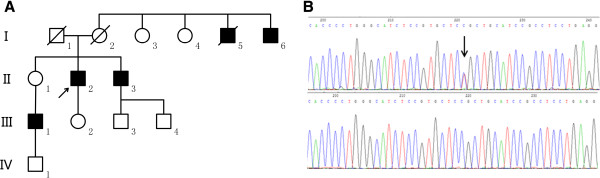
**Mutation Detection of** ***CACNA1S*** **in the Family of Hypokalaemic Periodic Paralysis**, **Type I** **(HOKPP1).** Panel **A** shows the family pedigree with the patient as the proband (arrow). Panel **B** shows the DNA sequencing results of *CACNA1S* of the patient and a healthy control and the position of the c.1582C > T heterozygous mutation (arrow). The mutation leads to an amino acid substitution of Arg for Cys at the 528th codon of CACNA1S.

Because of the presence of severe hypokalaemic periodic paralysis, the patient underwent a left adrenalectomy after admission. Examination of the adrenal gland revealed a 1.1-cm benign nodule at the periphery of the gland with multiple cortical nodular hyperplasias. The patient did not have any complications during the perioperative period and laboratory results were normal (serum potassium .87 mmol/L, serum sodium 143.9 mmol/L, norepinephrine 452 ng/L, adrenaline 60 ng/L, aldosterone 12.24 ng/dL, cortisol 8.1 μg/dL, renin 2.13 ng/mL, creatine kinase 60 IU/L, lactate dehydrogenase 168 IU/L, alanine aminotransferase 20 IU/L, aspartate transaminase 16 IU/L, creatinine 81.3 μmol/L, blood urea nitrogen 8.01 mmol/L). He was discharged 4 days after surgery. During the next 3 months, while recovering at home, no paralytic attacks occurred. The patient then returned to work, and the acute paralytic crises soon recurred. A colour Doppler ultrasound examination did not show any abnormality of the right adrenal gland. The patient then asked for help from the Department of Medical Genetics. His serum potassium was monitored three times with a frequency of once per month with results of 4.12, 3.97, and 4.27 mmol/L, respectively. As shown in Figure 1, in addition to the patient (II_2_), four other adult male family members, including two uncles (I_5_ and I_6_), one brother (II_3_), and one nephew (III_1_), also had a history of paralytic attacks, although their attacks were milder and less frequent (yearly to decadal). These members refused examination to determine the presence or absence of UAH.

Because of the positive family history of periodic paralysis with potential autosomal dominant inheritance, the diagnosis of HOKPP was considered, and genetic testing of the *CACNA1S* and *SCN4A* genes was performed using Sanger sequencing of all exons and their splice sites. Consequently, the patient was identified as a heterozygote carrying a novel missense mutation, c.1582C > T, in *CACNA1S* (p.Arg528Cys) (Figure 1). In his family, the mutation was also detected in three other adult males with periodic paralysis (I_6_, II_3_, and III_1_) and in two asymptomatic females (II_1_ and III_2_). This mutation was absent in two male family members who did not have a history of symptoms (III_3_ and III_4_). Furthermore, the targeted Sanger sequencing did not detect the mutation in 130 adult male controls.

During the following 2 years, the patient maintained the same diet as before surgery. He did not receive any potassium supplement treatment and suffered 12 paralytic attacks. However, there were considerably fewer attacks (monthly in the summer and no attacks in the winter), and the attacks were shorter in duration (2–3 hours) than the attacks before adrenalectomy.

## Discussion

Periodic paralysis caused by hypokalaemia possesses significant aetiological heterogeneity. A recent study [10] concluded that 42.9% of patients had a secondary cause, including renal tubular acidosis, Gitelman syndrome, thyrotoxicosis, alcoholism, hypothyroidism, Liddle’s syndrome, gastroenteritis, and primary hyperaldosteronism. In the other cases of primary periodic paralysis, 48.2% were sporadic and 8.9% had a positive family history [10]. Some of the cases with a positive family history might be attributed to mutations in a single gene, such as *CACNA1S* or *SCN4A*.

*CACNA1S* and *SCN4A* encode the human skeletal muscle α1-subunit of a dihydropyridine-sensitive calcium channel and the α-subunit of a sodium channel, respectively [7, 8]. Previous studies have linked mutations in these genes to HOKPP. The mutant CACNA1S and SCN4A have a partial loss of function, leading to reduced calcium or sodium current density, which is followed by membrane depolarisation. Membrane depolarisation is coupled with the inflow of potassium into skeletal muscle cells, causing paroxysmal hypokalaemia and periodic paralysis [11, 12]. In the present case, genetic testing revealed a missense mutation in *CACNA1S*, resulting in p.Arg528Cys. This mutation had not been reported previously. A genotype-phenotype correlation was identified in the patient’s family by the cosegregation of the mutation with all of the affected adult male members and a higher penetrance in males. In all of the seven HOKPP1–causing mutations that have been reported, two different mutations involve the 528th codon of *CACNA1S*, including p.Arg528His and p.Arg528Gly [5, 13]. The arginine residue encoded by the wild-type 528th codon is highly conserved among different species from *C. elegans* to humans, and it is located in the critical voltage sensor region of the transmembrane segment of the calcium channel [13]. The above evidence combined with the absence of the mutation in the matched male controls strongly suggests that the Arg528Cys substitution is a novel dominant HOKPP1-causing mutation accounting for the onset of hypokalaemic periodic paralysis in this patient’s family.

The patient also had UAH identified by a CT scan and pathological examination. UAH possesses a pathogenic mechanism distinct from HOKPP; however, the clinical manifestations of the two diseases are similar when UAH causes PA followed by hypokalaemia and muscular paralysis [14]. UAH was previously considered a rare subtype of PA [15], whereas recent studies suggest that the contribution of UAH to PA might have been underestimated because high-resolution multi-slice CT and other new screening tests have shown more patients with PA to have UAH [2]. This finding is encouraging because PA caused by UAH has been confirmed to be surgically correctable by adrenalectomy with excellent long-term results [3]. In the present case, the patient did not display PA, but his clinical manifestation was the most severe of his family members with periodic paralysis based on attack frequency and duration. In addition, there was no remission although he was over 43 years old, which could not be completely explained by HOKPP1 alone. After adrenalectomy, the symptoms of the patient were significantly relieved, including increased serum potassium concentrations (*t* test, α = 0.05: *P* = 0.009) between crises and decreased paralytic attack frequency and duration. This observation suggests a close association between the decreased serum potassium caused by UAH and the severity of HOKPP and supports the hypothesis that adrenalectomy may be an effective treatment for relieving the symptoms of patients with both UAH and severe hypokalaemic paralysis resulting from some incurable genetic diseases such as HOKPP and Gitelman syndrome [10, 16].

## Conclusions

In this first reported case of unilateral adrenal hyperplasia in concurrence with hypokalaemic periodic paralysis, we identified a novel dominant mutation, p.Arg528Cys, of *CACNA1S* causing HOPPK, which is the third known pathogenic mutation involving the 528th code of *CACNA1S*. These results emphasise the importance of CACNA1S Arg528 in maintaining calcium channel function. The prognosis after adrenalectomy suggests that the presence of UAH might increase the severity of hypokalaemic paralysis caused by other diseases. Operative treatment may be a rational choice for relieving the symptoms of hypokalaemic paralysis by increasing serum potassium, even if the UAH does not cause primary aldosteronism or clinical hypokalaemia.

## Consent

This study was approved by the Ethics Committee of Clinical Trials and Biomedical Research, West China Hospital, Sichuan University. Written informed consent for reporting results of genetic testing was obtained from the patient and his seven family members. A copy of the written consent is available for review by the Editor of this journal.
